# Sb_2_S_3_ Thickness-Related Photocurrent and Optoelectronic Processes in TiO_2_/Sb_2_S_3_/P3HT Planar Hybrid Solar Cells

**DOI:** 10.1186/s11671-019-3157-x

**Published:** 2019-10-16

**Authors:** Fan Wu, Rajesh Pathak, Lan Jiang, Weimin Chen, Chong Chen, Yanhua Tong, Tiansheng Zhang, Ronghua Jian, Qiquan Qiao

**Affiliations:** 1 School of Sciences and Key Lab of Optoelectronic Materials and Devices, Huzhou University, Huzhou, 313000 China; 2 Center for Advanced Photovoltaics, Department of Electrical Engineering and Computer Sciences, South Dakota State University, Brookings, SD 57007 USA; 3 Henan Key Laboratory of Photovoltaic Materials and School of Physics and Electronics, Henan University, Kaifeng, 475004 China; 4 Department of Materials Chemistry, Huzhou University, Huzhou, 313000 China

**Keywords:** Solar cells, Sb_2_S_3_, Photocurrent, Optoelectronic processes

## Abstract

In this work, a comprehensive understanding of the relationship of photon absorption, internal electrical field, transport path, and relative kinetics on Sb_2_S_3_ photovoltaic performance has been investigated. The n-i-p planar structure for TiO_2_/Sb_2_S_3_/P3HT heterojunction hybrid solar cells was conducted, and the photon-to-electron processes including illumination depth, internal electric field, drift velocity and kinetic energy of charges, photo-generated electrons and hole concentration-related surface potential in Sb_2_S_3_, charge transport time, and interfacial charge recombination lifetime were studied to reveal the key factors that governed the device photocurrent. Dark *J–V* curves, Kelvin probe force microscope, and intensity-modulated photocurrent/photovoltage dynamics indicate that internal electric field is the main factors that affect the photocurrent when the Sb_2_S_3_ thickness is less than the hole diffusion length. However, when the Sb_2_S_3_ thickness is larger than the hole diffusion length, the inferior area in Sb_2_S_3_ for holes that cannot be diffused to P3HT would become a dominant factor affecting the photocurrent. The inferior area in Sb_2_S_3_ layer for hole collection could also affect the *V*_oc_ of the device. The reduced collection of holes in P3HT, when the Sb_2_S_3_ thickness is larger than the hole diffusion length, would increase the difference between the quasi-Fermi levels of electrons and holes for a lower *V*_oc_.

## Introduction

Sb_2_S_3_ has been increasingly utilized for solid thin-film solar cells because of its moderate bandgap of 1.7 eV and an absorption coefficient of 1.8 × 10^5^ cm^−1^ [1, 2]. Sb_2_S_3_ thin films can be prepared by various methods, including spray pyrolysis [3], electrodeposition [4], chemically deposition [5], and thermal vacuum evaporation technique [6]. In Sb_2_S_3_-based photovoltaic device, photoelectric conversion efficiency (PCE) has reached to 5.7–7.5% by improved technology and device design [1, 2, 7–10]. However, current efficiencies of solid-state devices still remain low compared to other photovoltaic devices, such as dye-sensitized solar cells [11] and perovskite solar cells [12]. At present, most of the works usually focus on finding the best technology to get better optoelectronic performance in solid-state devices [7–10, 13–15]. In this regards, it is imperative to study the photo-electronic processes in Sb_2_S_3_-based solar cells for guiding the device design and optimization. This includes a comprehensive understanding of the balance among absorption, internal electrical field, and transport path, and relative kinetics on Sb_2_S_3_ photovoltaic performance, which is important to guide the optimization of the Sb_2_S_3_-based hybrid solar cells. In this work, the conventional TiO_2_/Sb_2_S_3_/poly(3-hexylthiophene-2,5-diyl(P3HT) n-i-p device structure was used to study the charge carrier generation and dissociation dynamic processes for different thicknesses of Sb_2_S_3_.

**Fig. 1:**
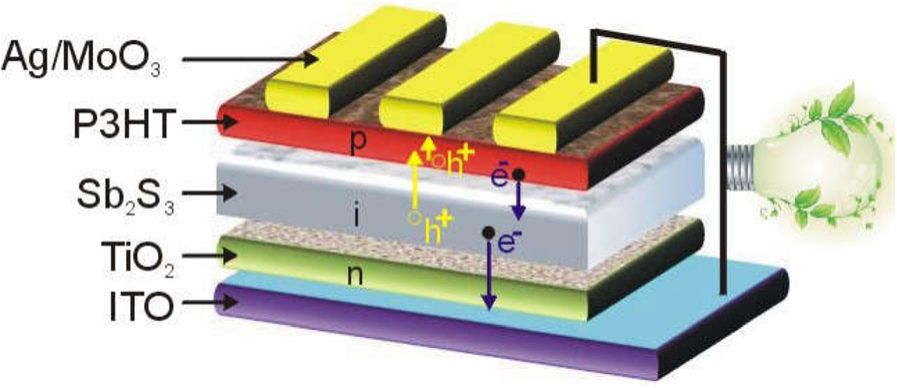
Illustration of TiO_2_/Sb_2_S_3_/P3HT n-i-p solar cell architecture.h^+^ denotes the hole and e^−^ denotes the electron

It is obvious that the different thickness of Sb_2_S_3_ in TiO_2_/Sb_2_S_3_/P3HT n-i-p solar cells can change (i) the amount of photon harvesting, which influences the photon-generated electron/hole concentration; (ii) the magnitude of internal electrical field across the Sb_2_S_3_ layer, which influences the photon-generated electron/hole drift; (iii) electron/hole transport distance to the respective electrode; and (iv) electron/hole recombination [16, 17]. However, the reason for the Sb_2_S_3_ thickness-dependent performance in n-i-p structure is still ambiguous, which has been simply attributed to the issues with bulk resistance, photon absorption, generation/recombination of charge carriers, and internal electric field [16–21], but the detailed and quantified analysis for the thickness-dependent photovoltaic parameters is not clear yet. To gain insight into the change of *J*_sc_ and *V*_oc_ upon the Sb_2_S_3_ thickness, TiO_2_/Sb_2_S_3_/P3HT n-i-p solar cells were fabricated (Fig. 1), and the thickness of Sb_2_S_3_-related photon-generated electron and hole transport processes which result in the different photocurrents was studied in this work. Moreover, we introduced dynamic intensity-modulated photocurrent/photovoltage spectra (IMPS/IMVS) and Kelvin probe force microscope (KPFM) characterization to study photon-to-electron processes and investigate the key factors that governed the device performance in different thicknesses of Sb_2_S_3_ solar cells.

## Methods

### Reagents

Etched FTO-coated glass substrates were purchased from Huanan Xiangcheng Co., Ltd., China. SbCl_3_ (99%), Na_2_S_2_O_3_ (99%), and titanium diisopropoxide (75% in isopropyl alcohol) were purchased from Adamas-beta. P3HT was ordered from Xi’an Polymer Company, China, and Ag (99.999%) was ordered from Alfa.

### Device Fabrication

The substrates were cleaned via ultrasonication in soap water, acetone, and isopropanol for 60 min each, followed by treatment with UV-ozone for 30 min. A thin layer of compact TiO_2_ (0.15 M titanium diisopropoxide in ethanol) was spin coated at 4500 rpm for 60 s, followed by annealing at 125 °C for 5 min and 450 °C for 30 min. Deposition of Sb_2_S_3_ on the top of the TiO_2_ thin layer was performed by a chemical bath deposition (CBD) method [5, 10, 22]. An acetone solution containing SbCl_3_ (0.3 M) was added dropwise into Na_2_S_2_O_3_ (0.28 M) with stirring in an ice bath (~ 5 °C). The FTO substrate was covered with a thin layer of TiO_2_ and then suspended upside-down in the aqueous solution when the color of the solution changed to orange. After 1 h, 1.5 h, 2 h, and 3 h of the CBD process, a smooth and uniform amorphous Sb_2_S_3_ layer was deposited onto the TiO_2_-coated FTO substrates, and the sample was thoroughly rinsed with de-ionized water and dried under N_2_ flow. The substrate was further annealed for 30 min in a glovebox (O_2_: 0.1 ppm, H_2_O: 0.1 ppm) under an N_2_ atmosphere. The fabrication of n-i-p heterojunction was completed by spin casting (1500 rpm for 60 s) of P3HT (15 mg/mL) film on top of Sb2S3 inside a glovebox (O_2_: 0.1 ppm, H_2_O: 0.1 ppm) under an N_2_ atmosphere. Finally, the MoO_3_ (10 nm) and Ag (100 nm) electrode was deposited by evaporation through a shadow mask.

### Instruments and Characterization

X-ray diffraction (XRD) patterns of the film were recorded by an MXP18AHF X-ray diffractometer with Cu Kα irradiation (*λ* = 1.54056 Å). Scanning electron microscopy (SEM) measurements were performed on a field-emission scanning electron microscope (ZEISS, GeminiSEM 300). The absorption spectra were recorded with a Shimadzu UV-2600 spectrophotometer. Current density–voltage (*J*–*V*) characteristics were measured under AM 1.5 illumination with an intensity of 100 mW/cm^2^ using a 94023A Oriel Sol3A solar simulator (Newport Stratford, Inc.). The light intensity from a 450 W xenon lamp was calibrated with a standard crystalline silicon solar cell. The *J–V* curves were collected using an Oriel I–V test station (Keithley 2400 Source Meter, Newport). External quantum efficiency (EQE) spectra of the solar cells were measured by using a QE/IPCE measurement kit (Zolix Instruments Co., Ltd.) in the spectral range of 300–900 nm. Intensity-modulated photocurrent spectra (IMPS) and intensity-modulated photovoltage spectra (IMVS) were measured using an electrochemistry workstation (IviumStat.h, Netherlands) under ambient conditions with a background intensity of 28.8 mW/cm^2^ from a white light-emitting diode, with a small sinusoidal perturbation depth of 10%. Kelvin probe force microscope (KPFM) was performed by an Agilent SPM 5500 atomic force microscope equipped with a MAC III controller (comprising three lock-in amplifiers) to map the surface potential (SP).

## Results and Discussion

### Deposition and Characterization of Sb_2_S_3_/TiO_2_ Film

**Fig. 2:**
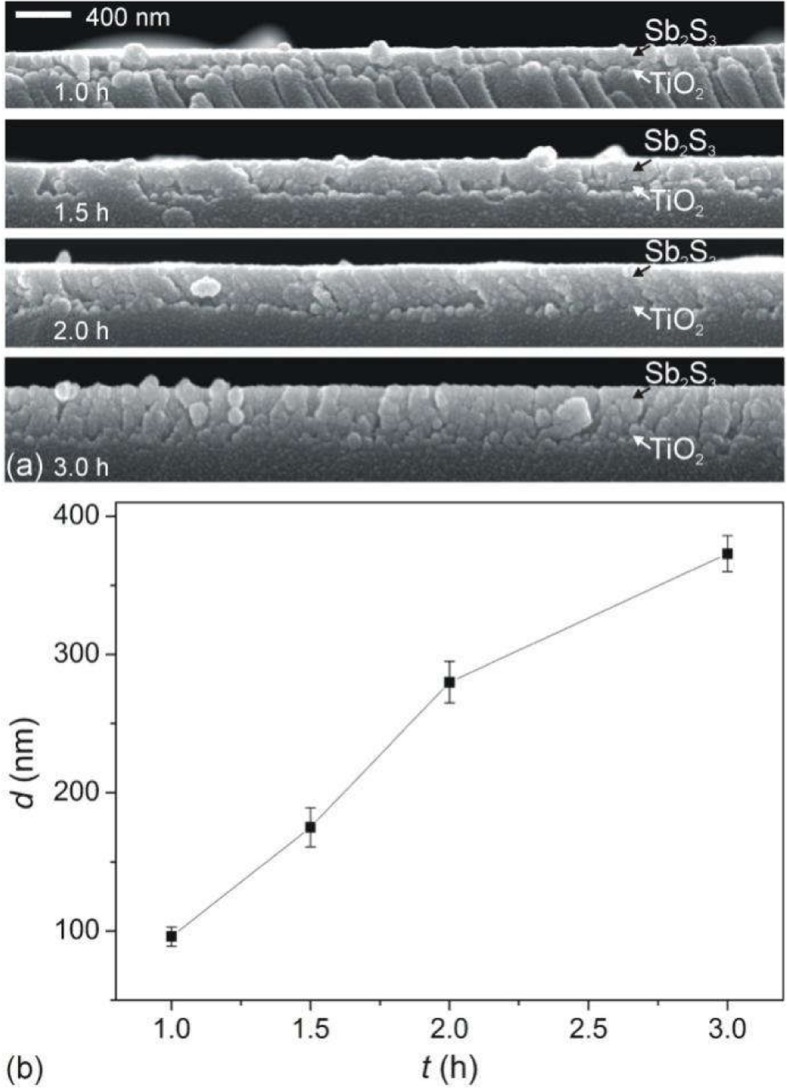
**a** Cross-sectional FE-SEM images of Sb_2_S_3_ films on TiO_2_ dense layer-coated glass substrates. **b** Average Sb_2_S_3_ thickness *d* plotted as a function of the CBD reaction time *t* for the Sb_2_S_3_ film deposition. The values were estimated by the FE-SEM cross-section images

**Fig. 3:**
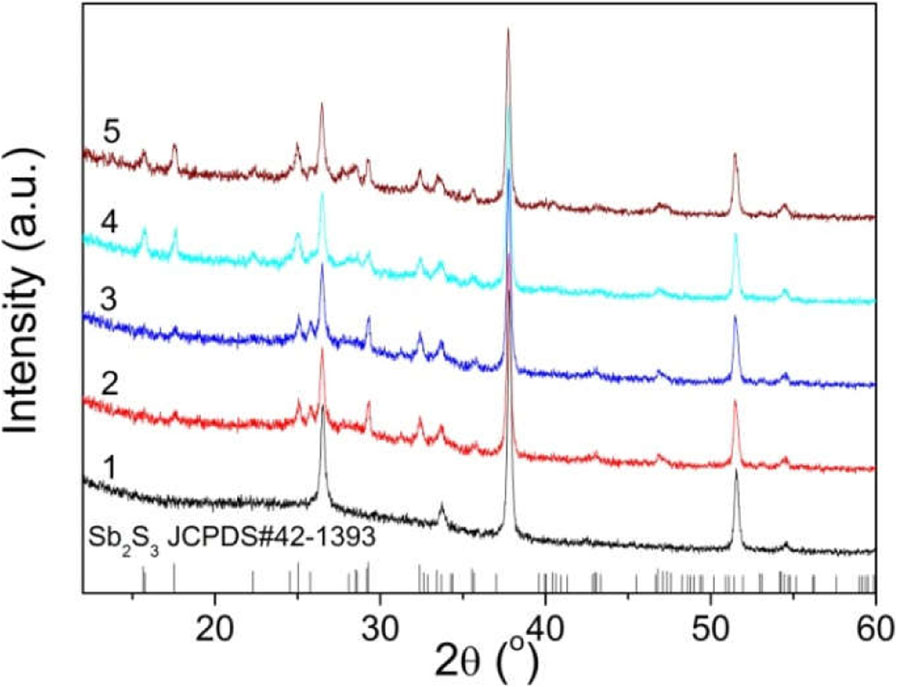
XRD patterns of the as-synthesized Sb_2_S_3_ film on FTO with different deposition time. Sample 1 is the pure FTO glass substrate, and samples 2–5 are Sb_2_S_3_ films with *t* of 1 h, 1.5 h, 2 h, and 3 h, respectively

FE-SEM images (Fig. 2a) clearly show that different thicknesses of Sb_2_S_3_ film are deposited on TiO_2_ layer-coated glass substrates with the different CBD time *t* (1.0 h, 1.5 h, 2.0 h, 3.0 h). It can be seen that the uniform Sb_2_S_3_ layers were successfully obtained by CBD techniques. The average thickness of the Sb_2_S_3_ film estimated from the cross-sectional FE-SEM images is plotted in Fig. 2b as a function of the CBD time. The average thickness *d* of Sb_2_S_3_ film increases linearly with *t* (Fig. 2b). The average thickness increased almost linearly from 96 to 373 nm by changing CBD time from 1 to 3 h. The XRD patterns of Sb_2_S_3_ film with different thickness of Sb_2_S_3_ film on FTO glass are shown in Fig. 3. The measured XRD spectrum is indexed to orthorhombic Sb_2_S_3_ (JCPDS PCPDFWIN #42-1393) [23].

**Fig. 4:**
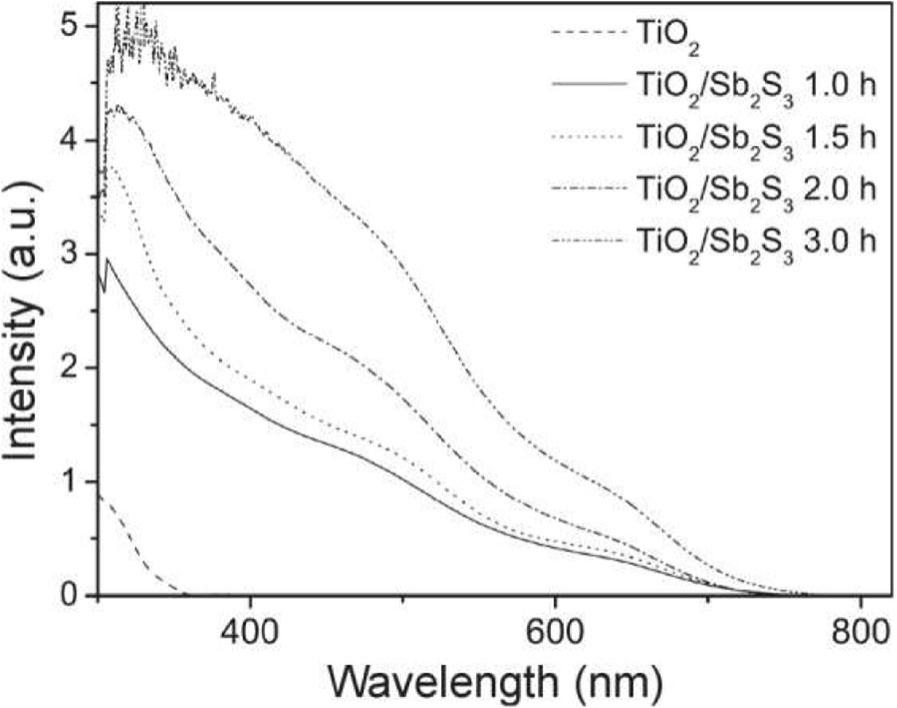
The UV-vis absorption of TiO_2_ and TiO_2_/Sb_2_S_3_ films with *t* of 1–3 h, respectively

As shown in Fig. 4, the TiO_2_ samples exhibit the absorption onset at 386 nm (3.21 eV) corresponding to the bandgap absorption of TiO_2_ [24]. All the as-deposited TiO_2_/Sb_2_S_3_ layers with different *t* of CBD exhibit an absorption edge at ca. 750 nm [25]. The absorption intensity of Sb_2_S_3_ on the TiO_2_ surfaces is clearly in the order 3 h > 2 h > 1.5 h > 1 h. This result also indicates that the Sb_2_S_3_ film gradually becomes thicker with a longer CBD *t*, which also agrees with the SEM results.

### Solar Cells

**Fig. 5:**
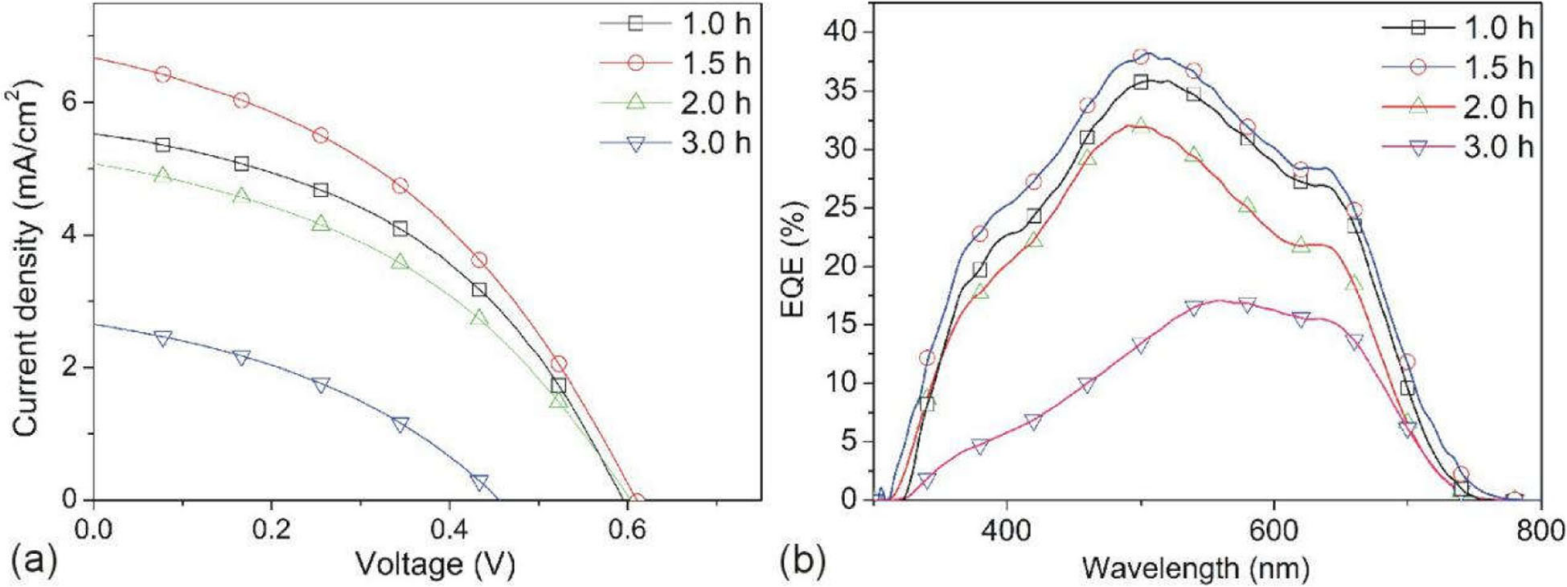
**a** *J–V* curves and **b** EQE spectra of the solar cells with different CBD *t* for Sb_2_S_3_ film

*J*-*V* characteristics of solar cells with different thickness *d* (i.e., CBD *t*) are compared in Fig. 5a. Table 1 presents the overall photovoltaic performance of these devices. Increasing thickness *d* (i.e., CBD time *t*) significantly affects device performance. The PCE increases as *d* increases from 96 to 175 nm (i.e., *t* increased from 1.0 to 1.5 h) and decreases thereafter, especially decreases largely after *d* > 280 nm (i.e., *t* > 2 h). Optimum Sb_2_S_3_ thickness of 175 nm can be determined by comparison of device efficiencies, at which point a maximum PCE of 1.65%, *J*_sc_ of 6.64 mA cm^−2^, *V*_oc_ of 0.61 V, and FF of 40.81% can be achieved. This result is comparable to the other’s reports [16, 26]. Liu et al. studied hybrid ZnO/Sb_2_S_3_/P3HT n-i-p cells with Sb_2_S_3_ layers of three different thickness (50, 100, and 350 nm) by thermal evaporation achieving the highest PCE (~ 2%) with the 100-nm-thick Sb_2_S_3_ [12]. Kamruzzaman et al. studied TiO_2_/Sb_2_S_3_/P3HT n-i-p cells with Sb_2_S_3_ thicknesses of 45–120 nm by a thermal evaporation method, and the absorber Sb_2_S_3_ and hole transporting layer P3HT were annealed under atmospheric conditions. In their studies, the thickness of 100–120 nm showed a better power conversion efficiency of 1.8–1.94% [26]. Obviously, the thickness of Sb_2_S_3_ indeed strongly affects the device performance, even by different deposition strategies of Sb_2_S_3_ film or annealing condition.

**Table 1:** Device performance of the TiO_2_/Sb_2_S_3_/P3HT n-i-p planar solar cells under AM 1.5 illumination of 100 mW/cm^2^

CBD time (h)	Thickness *d* (nm)	*V*_oc_ (*V*)	*J*_sc_ (mA cm^−2^)	FF (%)	PCE (%)	*τ*_IMPS_ (μs)	*τ*_IMVS_ (ms)
1.0	96	0.59	5.50	43.93	1.44	5.04	1.01
1.5	175	0.61	6.64	40.81	1.65	5.52	1.01
2.0	280	0.60	5.06	40.87	1.25	6.34	1.01
3.0	373	0.46	2.64	37.56	0.45	6.64	1.01

### Charge Transport

**Fig. 6:**
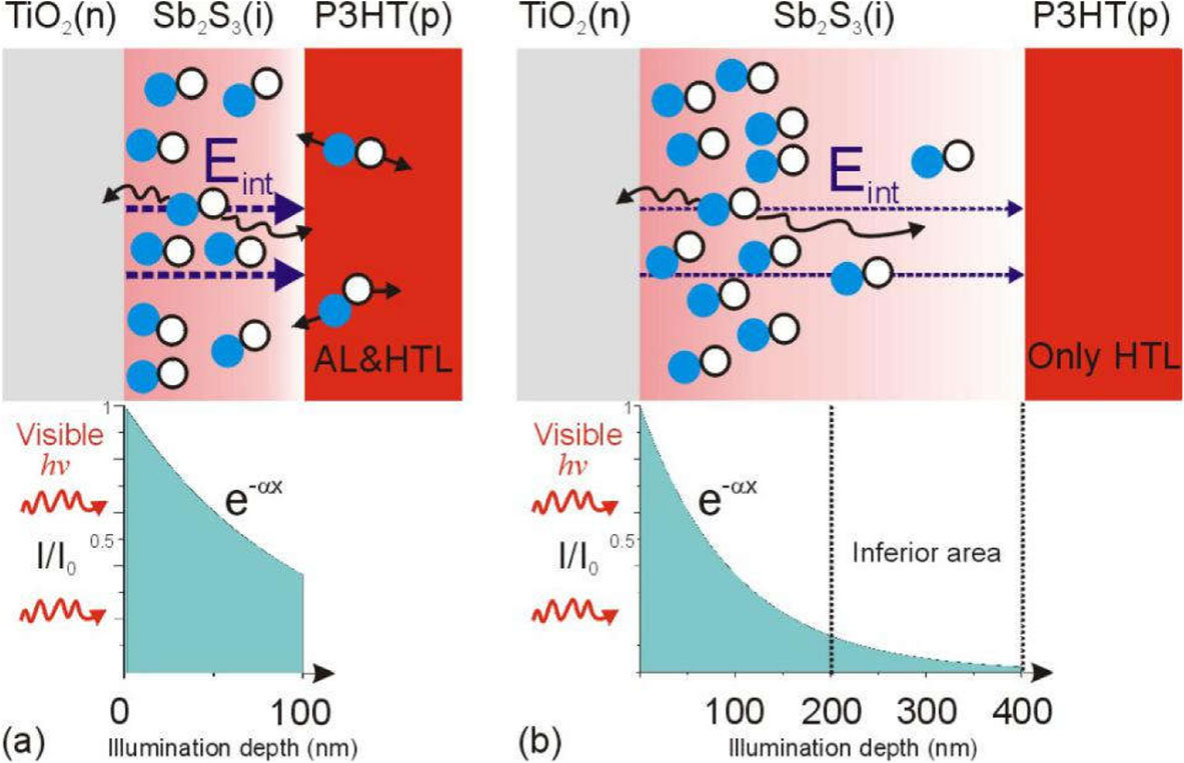
The illustration of the Sb_2_S_3_ thickness *d*-dependent illumination depth *x* and *E*_in_

**Fig. 7:**
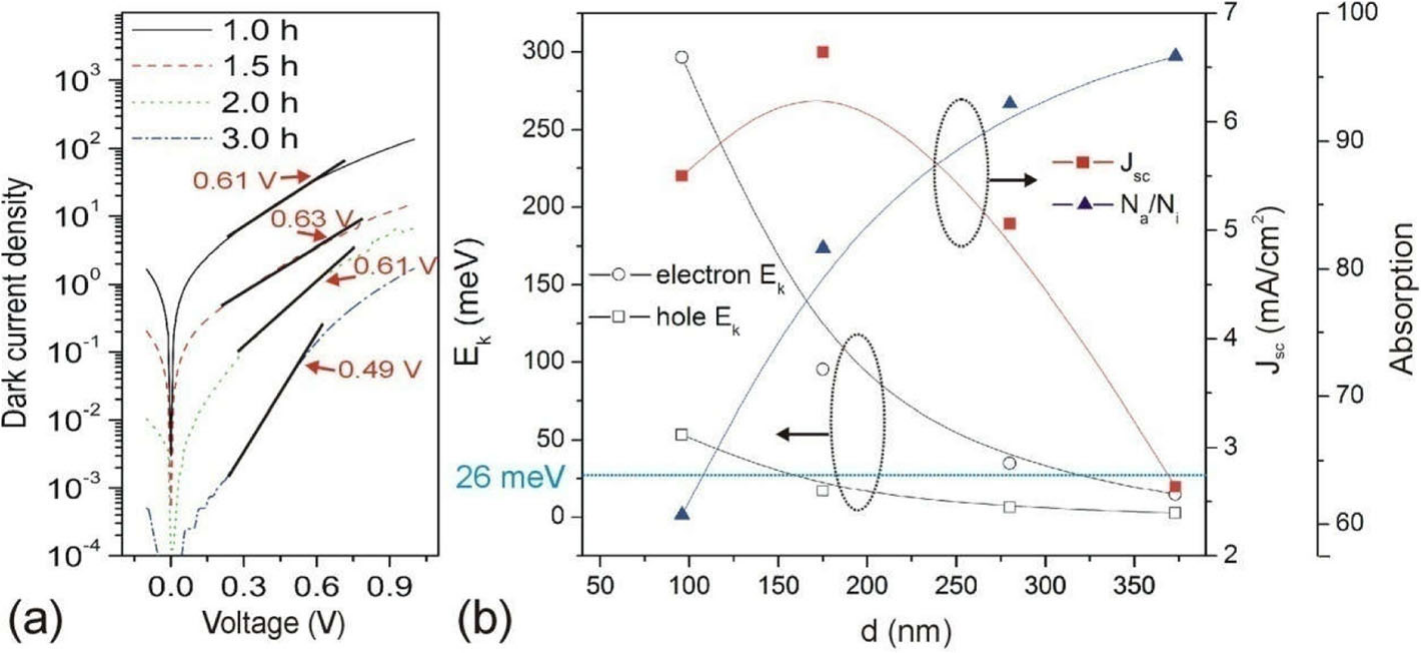
**a** Semilogarithmic plots of *J–V* characteristic in the dark of the solar cells with different CBD *t* for Sb_2_S_3_ film. **b** Dependences of *V*_in_, *J*_sc_, *N*_a_/*N*_i_, *E*_ke_, and *E*_kh_ on Sb_2_S_3_ thickness *d*

The device *J*_sc_ increases remarkably with increasing Sb_2_S_3_ thickness *d* from 96 to 175 nm and then decreases as the *d* increases (Fig. 5 and Table 1). The device *J*_sc_ is significantly dependent on the Sb_2_S_3_ thickness *d.* The charge carrier generation and dissociation are key processes for photocurrent generation. Firstly, visible light will pass through the TiO_2_ layer due to its visible light window property (Fig. 4) and begin to be absorbed from TiO_2_/Sb_2_S_3_ interface. Sb_2_S_3_ has been proved to be a high absorption coefficient *α* around 10^5^ cm^−1^ in the visible region [27]. Here, we take *α* = 10^5^ cm^−1^ for Sb_2_S_3_. The thickness-dependent illumination depth is depicted in Fig. 6 according to the Beer-Lambert law *I*(*x*) = *I*_0_ e^-ax^, in which the *I*_0_ is the incident photon flux and the *I*(*x*) is the photon flux in Sb_2_S_3_. Obviously, the incident photons cannot be absorbed fully when the Sb_2_S_3_ has a thickness of 100 nm or 200 nm (Fig. 6b). The *d*-related ratio of absorbed photon (N_a_)/incident photons (N_i_) can be calculated by integration of the area of the shaded area in coordinate. As shown in Fig. 6b (also refer Fig. 7b), the N_a_/N_i_ is 61% when the *d* = 96 nm and N_a_/N_i_ is enhanced to 82% when the *d* = 175 nm. It can be believed that the further 21% photons absorbed might cause the increase in *J*_sc_ from 5.50 to 6.64 mA/cm^2^. When the *d* increases to 280 nm, the extra 11% photons are absorbed and the N_a_/N_i_ is further enhanced to 93%, which shows that more photons could be further absorbed and then might generate more electrons. However, the device *J*_sc_ decreased to 5.06 mA/cm^2^ which is lower than the case of *d* = 96 nm. When the *d* increases to 373 nm, the N_a_/N_i_ is close to 100%, and the device *J*_sc_ is sharply decreased to 2.64 mA/cm^2^. Therefore, absorption is not the sole factor that affects *J*_sc_.

The semilogarithmic plots of the *J–V* curve of solar cells in the dark normally exhibit three distinct regimes: **(**i) linear increase for leakage dominated current, (ii) exponential increase for diffusion dominated current, and (iii) quadratic increase for space-charge-limited current. The built-in voltage (*V*_in_) normally can be estimated at the turning point where the dark curve begins to follow a quadratic behavior (Fig. 7a). Dependences of *V*_in_, *J*_sc_, *N*_a_/*N*_i_, *E*_ke_, and *E*_kh_ on CBD *t* are shown in Fig. 7b. When *d* increased from 96 to 175 nm, the *N*_a_/*N*_i_ enhanced 34.44%; however, the *J*_sc_ only increased by 20.72%, which means that there is another factor limiting the *J*_sc_ increment. It has been inferred that this was might due to the decreased internal electrical field across the Sb_2_S_3_ layer, which weakened the photon-generated electron/hole drift [16]. Therefore, we calculated the internal electrical field *E*_in_ cross the Sb_2_S_3_ based on the relation of *E*_in_ = *V*_in_/*d* (Table 2). Moreover, the drift velocity of electron *v*_e_ and hole *v*_e_, kinetic energy of electron *E*_ke_, and hole *E*_kh_ under internal electric field *E*_in_ were also calculated (Table 2 and Fig. 7b). When the *d* is 96 nm, the *E*_ke_ is 296.56 meV, and *E*_kh_ is 53.25 meV. When the *d* increased to 175 nm, the *E*_ke_ largely decreases to 95.29 meV and *E*_kh_ decreased to 17.12 meV, which is lower than the thermal energy at ambient temperature (*E*_kt_, 26 meV). This result indicates that the internal electric field has little effects on hole drift when Sb_2_S_3_ thickness is or larger than 175 nm. Obviously, the reduced *E*_ke_ and *E*_kh_ with the thicker Sb_2_S_3_ should be the reason that limits the increment of *J*_sc_. Further increasing *d* from 175 to 280 nm, the *N*_a_/*N*_i_ enhanced to 13.84%; however, the *J*_sc_ get decreased. This might be due to the decrease in *E*_ke_ which is close to the *E*_kt_ (*d* = 280 nm) but much lower than the *E*_kh_ (*d* = 373 nm), which means the *E*_in_ gradually has little effects on electron drift when *d* > 280 nm as observed in this work. Therefore, *E*_in_ decrement-related electron drift might be responsible for the *J*_sc_ reduction when *d* increased from 175 to 280 nm. However, when the *d* increased to 373 nm, the *E*_in_ has little effects on electron and hole drift, but *J*_sc_ still largely decreased, which indicates that *E*_in_ is also not the sole factor that affects the *J*_sc_.

**Table 2:** Parameters of *V*_in_, internal electric field *E*_in_, drift velocity of the electron (*v*_e_) and hole (*v*_h_), and kinetic energy of the electron (*E*_ke_) and hole (*E*_kh_) in the dark of the solar cells with different CBD *t*

*d* (nm)	*V*_in_ (V)	*E*_in_ (× 10^4^ Vcm^−1^)	*v*_e_ (cm s^−1^)	*E*_ke_ (meV)	*v*_h_ (cm s^−1^)	*E*_kh_ (meV)
96	0.61	6.35	42.55	296.56	16.51	53.25
175	0.63	3.60	24.12	95.29	9.36	17.12
280	0.61	2.18	14.61	34.96	5.67	6.28
373	0.49	1.31	8.78	12.63	3.67	2.26

The mean value of *d* for a certain Sb_2_S_3_ deposition time *t* is used for calculation of the above parameters *E*_in_,*v*_e,_*v*_h,_ *E*_ke_, and *E*_kh_

**Fig. 8:**
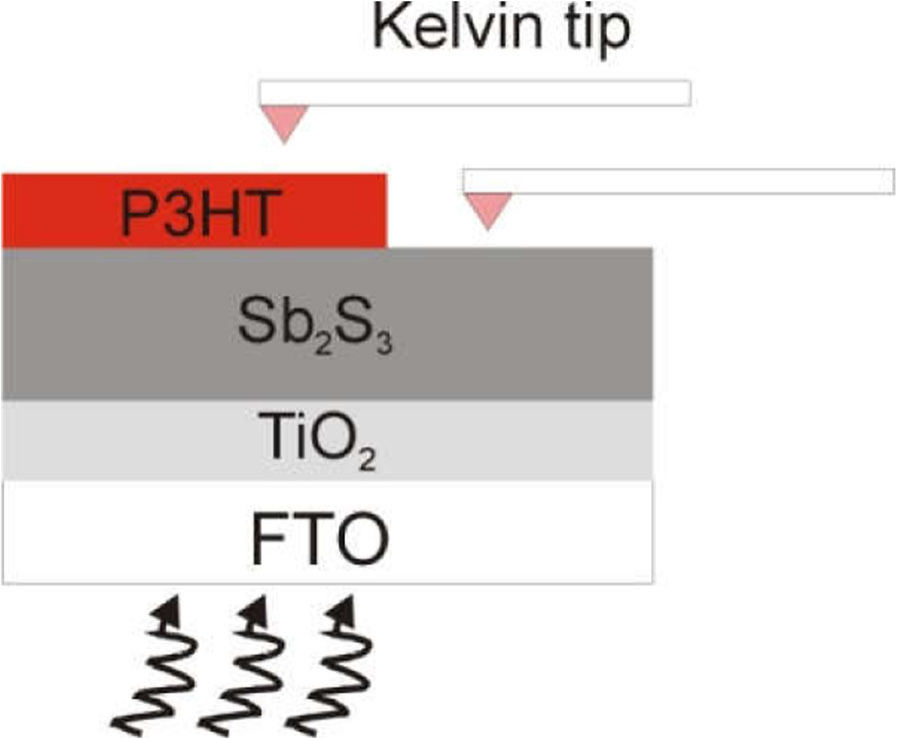
Illustration of SP measurement of Sb_2_S_3_/P3HT interface by KPFM

We used KPFM to characterize the photo-generated electrons and hole concentration-related surface potential (SP) in Sb_2_S_3_/P3HT. The sample for the KPFM measurement was prepared by drop casting the P3HT precursor solution onto part of the FTO/TiO_2_/Sb_2_S_3_ film surface (Fig. 8). As the Sb_2_S_3_ thickness increases from 96 to 373 nm, the SP on the top of Sb_2_S_3_ gradually becomes smaller, which means the Fermi level on the Sb_2_S_3_ surface becomes lower [28]. This demonstrates that electrons which could diffuse to the top surface are being gradually reduced, indicating that there is an inferior region for photo-generated electrons in thicker Sb_2_S_3_ film as shown in Fig. 6. We also examined the SP of P3HT part. The changes of SP of the P3HT are different from that of Sb_2_S_3_. P3HT might be excited by light to generate excitons and then separate into electrons and holes [29, 30], when Sb_2_S_3_ is very thin (< 200 nm). When Sb_2_S_3_ becomes thicker, P3HT only acts as the hole transport layer, because most of the photons are absorbed by Sb_2_S_3_ (Fig. 3). Therefore, when the thickness of Sb_2_S_3_ is less 280 nm, P3HT could be photo-excited, resulting in the Fermi level of P3HT gradually decreases as Sb_2_S_3_ thickness gradually increases (decreased photo-exciton). In the case of 280 nm, the SP of P3HT drops rapidly, because there is no photo-exciton and the P3HT works just as a hole transport layer to collect holes. As the Sb_2_S_3_ thickness increases to 373 nm which is much larger than the hole transport length, the hole collection also drops rapidly, causing the Fermi level in P3HT to rise again. Moreover, the changes of SP in P3HT is much larger than that in the Sb_2_S_3_ in the case of *d* = 373 nm, which means that hole collection is worse than electron collection and therefore would probably lead to a much decreased *J*_sc_.

Furthermore, IMPS and IMVS, as the powerful dynamic photoelectrochemical methods in dye-sensitized solar cells [31] and perovskite solar cells [32], have been applied to study the charge transport dynamics in this work. IMPS/IMVS measures the photocurrent/photovoltage response to a small sinusoidal light perturbation superimposed on the background light intensity under short-circuit/open-circuit condition [31–33]. The measured IMPS or IMVS responses appear in the fourth quadrant of the complex plane with a shape of the distorted semicircle (Fig. 10a, b). The time constant *τ* defined by the frequency (*f*min) of the lowest imaginary component of IMPS or IMVS response is an evaluation of the transit time *τ*_IMPS_ for the electrons to reach the collection electrode under short-circuit condition or the electron lifetime *τ*_IMVS_ related to interfacial charge recombination under open-circuit condition. According to the relation *τ* = (2π*f*)^−1^ [31–35], *τ*_IMPS_ and *τ*_IMVS_ in the devices were calculated (Table 1). The increased *τ*_IMPS_ suggests a longer transport path of charges to collection electrode, whereas the unchanged *τ*_IMVS_ infers the same interfacial charge recombination [33]. The interfacial charge collection efficiency *η*_c_ is typical considered as *η*_c_ = 1-*τ*_IMPS_/*τ*_IMVS_ [31–35]. Obviously, the longer transport time of the *τ*_IMPS_ and the short interfacial charge recombination lifetime of the *τ*_IMVS_ would cause a worse charge collection and vice versa. In this study, the *τ*_IMPS_ increases with the thicker Sb_2_S_3_ while the *τ*_IMVS_ is unchanged. Therefore, interfacial charge collection efficiency *η*_c_ decreases with the thicker Sb_2_S_3_, and the changes of *J*_sc_ in different thickness of Sb_2_S_3_ solar cells should be caused by the transport path and charge collection efficiency, not by charge recombination.

**Fig. 9:**
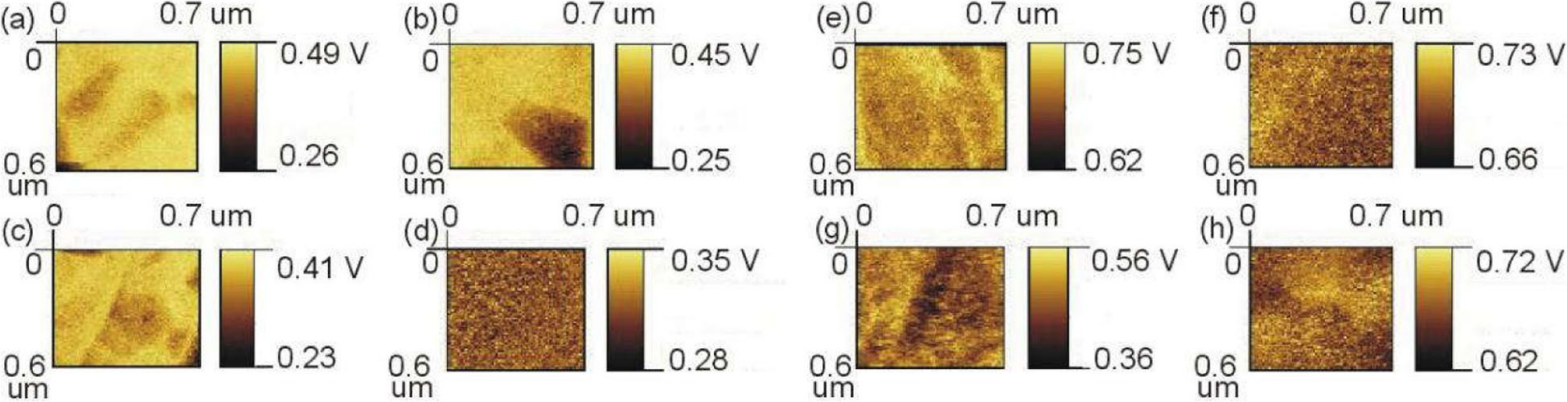
KPFM images of Sb_2_S_3_ of 1 h (**a**), 1.5 h (**b**), 2 h (**c**), and 3 h (**d**) and P3HT on Sb_2_S_3_ of 1 h (**e**), 1.5 h (**f**), 2 h (**g**), and 3 h (**h**) under white light illumination from FTO glass, respectively. **i**, **j** The corresponding SP distributions of Sb_2_S_3_ and P3HT

**Fig. 10:**
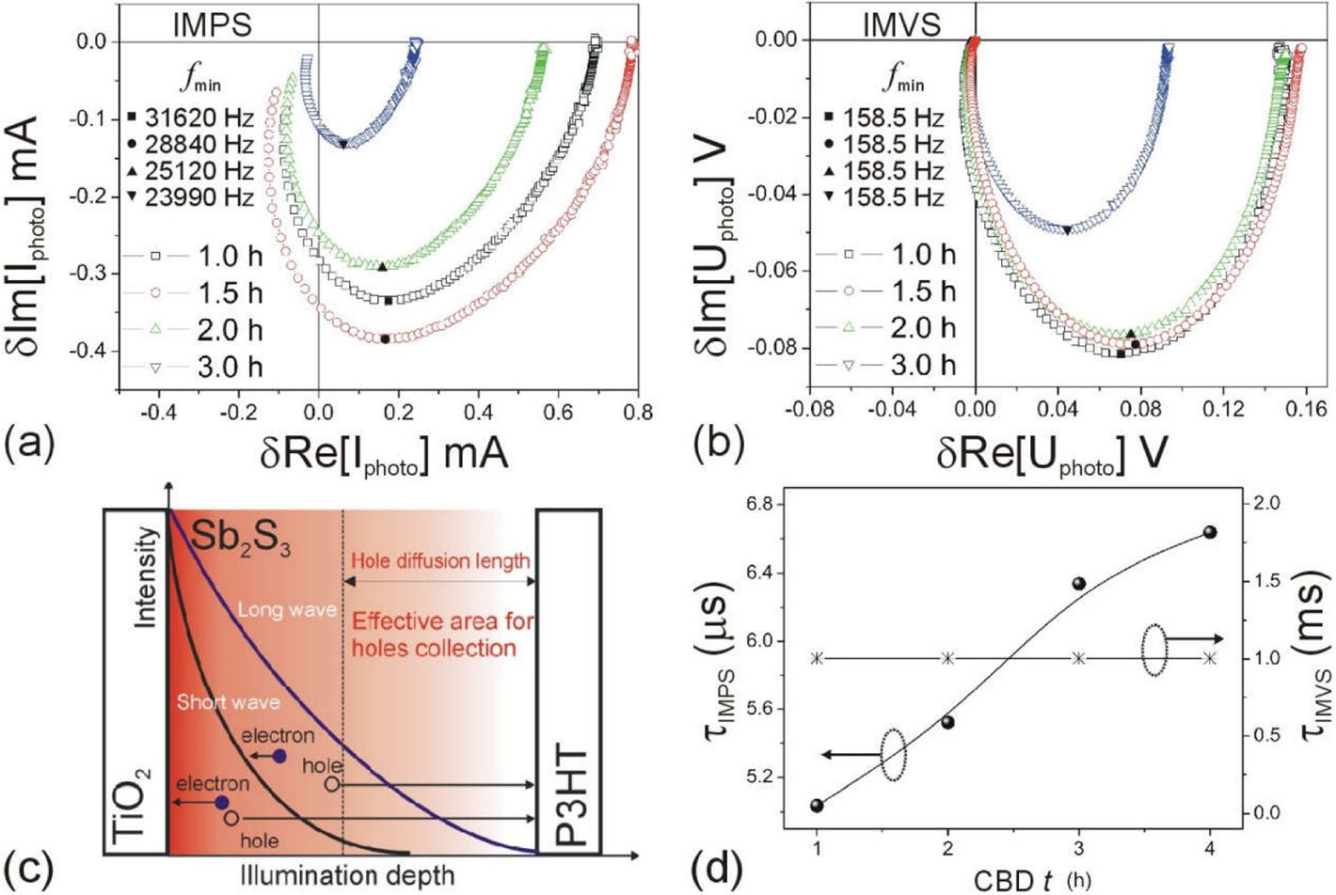
**a** IMPS and **b** IMVS characterizations of solar cells with different CBD *t* for Sb_2_S_3_ film. **c** Illustration of electron and hole diffusion area for short and long wavelength illumination. **d** Dependence of *τ*_IMPS_ and *τ*_IMVS_ on CBD *t*

The increase in Sb_2_S_3_ thickness could absorb more photons which could enhance the photocurrent. However, in thicker Sb_2_S_3_ layer, most of electrons and holes are generated near the TiO_2_ side due to exponential photon absorption (Fig. 10c); therefore, the transport path of most of the electrons are almost the same. However, most of the holes need to be diffused in a longer path than electrons in the thicker Sb_2_S_3_ layer, which is demonstrated by longer *τ*_IMPS_ in Fig. 10d. When the thickness exceeds the hole diffusion length, the inferior area in Sb_2_S_3_ for an inefficient hole generation and transport would decrease the photocurrent and weaken the *J*_sc_ and EQE. The hole diffusion length in Sb_2_S_3_ is around 180 nm [18]. When the thickness of Sb_2_S_3_ exceeds hole diffusion length, the collection performance of holes is reduced which is also responded by EQE spectra (Fig. 5b) since the absorption coefficient of the long wave is much lower than the short wave, resulting in a longer illumination depth for long wave (Fig. 9) [35]. Photo-generated holes from long band could distribute more uniform in Sb_2_S_3_ than that from short band (photo-generated holes from short band could close to TiO_2_ side), resulting in a more efficient collection of the hole from long band. Therefore, the EQE in long-wavelength part did not get a large decreased as much as short-wave part with Sb_2_S_3_ thickness of 373 nm (Fig. 5b).

As shown in Fig. 10d, it is easily understood that a smaller *τ*_IMPS_ is accompanied by a thinner Sb_2_S_3_ (i.e., a shorter charge transport path); however, *τ*_IMVS_ mainly remains the same when Sb_2_S_3_ thickness increased from 96 to 373 nm in this experiment, which means that there is no direct dependence of *J*_sc_ and *V*_oc_ on *τ*_IMVS_ (i.e., interfacial recombination) when Sb_2_S_3_ thickness changes. It is well known that the *V*_oc_ of the TiO_2_/Sb_2_S_3_/P3HT solar cells is normally determined by the difference between the quasi-Fermi levels of the electrons in the TiO_2_ and the holes in the P3HT [36]. As the collection of holes is reduced in P3HT when the thickness of Sb_2_S_3_ is larger than the hole diffusion length, it would increase the difference between the quasi-Fermi levels of electrons and holes for a lower *V*_oc_. In addition, a thicker Sb_2_S_3_ would increase the higher series resistance and worse charge collection efficiency; these unfavorable factors may cause a lower FF in thicker Sb_2_S_3_ device.

Although, the efficiency of planar TiO_2_/Sb_2_S_3_/P3HT n-i-p solar cells is very low, and how to further improve the device efficiency is a challenge. However, our results still demonstrated that some further improvements could be carried out. For example, enhancing the built-in electric field by employing some different electron transport layer or hole transport layer could enhance charge transport and collection. Moreover, how to improve the hole diffusion ability should be considered; maybe some conductive additives is helpful. In addition, interfacial engineer is also important for improving charge transfer and dissociation. Last but not least, the method that expressed in this paper might be offering some helpful reference for other relative high-efficiency solar cells (e.g., organic solar cells, perovskite solar cells).

## Conclusion

In this paper, the mechanism of photocurrent changes in TiO_2_/Sb_2_S_3_/P3HT n-i-p solar cells with different thickness of Sb_2_S_3_ was studied. When the thickness is less than the hole transport length, the absorption and internal electric field are the main factors that affect the photocurrent; when the thickness is larger than the hole transport length, the inferior area in Sb_2_S_3_ for an inefficient hole generation and transport is the main reason for photocurrent decrement. Results showed that device short-circuits’ current density (*J*_sc_) is increased with the enhanced photon absorption when the Sb_2_S_3_ thickness is less than the hole transport length; however, when the Sb_2_S_3_ thickness is larger than the hole transport length, device *J*_sc_ is sharply decreased with further increased absorption. Internal electric field decrement-related electron drift could lead to the reduction in the *J*_sc_ when the thickness of Sb_2_S_3_ is less than the hole transport length. However, when the thickness of Sb_2_S_3_ is larger than the hole transport length, the internal electric field has little effects on electron and hole drift, but *J*_sc_ still largely decreased. KPFM and IMPS/IMVS characterization demonstrated that there is an inferior region for photo-generated electrons in thicker Sb_2_S_3_ film. The inferior area in Sb_2_S_3_ for a reduction of holes that can diffuse into the P3HT when the Sb_2_S_3_ thickness is larger than the hole diffusion length, leading to the obviously decreased *J*_sc_. Moreover, the reduced collection of holes in P3HT with the increased thickness of Sb_2_S_3_ would increase the difference between the quasi-Fermi levels of electrons and holes for a lower *V*_oc_.

## Abbreviations

CBD: Chemical bath deposition
*E*_in_: Internal electric field
*E*_ke_: Kinetic energy of the electron
*E*_kh_: Kinetic energy of the hole
*E*_kt_: Thermal energy at ambient temperature
EQE: External quantum efficiency
FF: Fill factor
IMPS: Intensity-modulated photocurrent spectra
IMVS: Intensity-modulated photovoltage spectra
*J*–*V*: Current density–voltage
*J*_sc_: Short-circuit current
KPFM: Kelvin probe force microscope
N_a_: Absorbed photon
N_i_: Incident photons
P3HT: Poly (3-hexylthiophene-2,5-diyl
PCE: Photoelectric conversion efficiency
SEM: Scanning electron microscopy
SP: Surface potential
UV-vis: Ultraviolet-visible spectroscopy
*v*_e_: Drift velocity of the electron
*v*_h_: Drift velocity of the hole
*V*_in_: Built voltage
*V*_oc_: Open-circuit voltage
XRD: X-ray diffraction
